# Squeezing out the last egg—annual fish increase reproductive efforts in response to a predation threat

**DOI:** 10.1002/ece3.3422

**Published:** 2018-06-07

**Authors:** Arnout Francis Grégoir, Eli Samuel Joachim Thoré, Charlotte Philippe, Tom Pinceel, Luc Brendonck, Bram Vanschoenwinkel

**Affiliations:** ^1^ Animal Ecology, Global Change and Sustainable Development University of Leuven Leuven Belgium; ^2^ Systemic Physiological and Ecotoxicological Research University of Antwerp Antwerp Belgium; ^3^ Centre for Environmental Management University of the Free State Bloemfontein South Africa; ^4^ Research Unit for Environmental Sciences and Management North‐West University Potchefstroom South Africa; ^5^ Community Ecology Laboratory Department of Biology Vrije Universiteit Brussel Brussels Belgium

**Keywords:** life history, *Nothobranchius*, phenotypic plasticity, predation risk

## Abstract

Both constitutive and inducible antipredator strategies are ubiquitous in nature and serve to maximize fitness under a predation threat. Inducible strategies may be favored over constitutive defenses depending on their relative cost and benefit and temporal variability in predator presence. In African temporary ponds, annual killifish of the genus *Nothobranchius* are variably exposed to predators, depending on whether larger fish invade their habitat from nearby rivers during floods. Nonetheless, potential plastic responses to predation risk are poorly known. Here, we studied whether *Nothobranchius furzeri* individuals adjust their life history in response to a predation threat. For this, we monitored key life history traits in response to cues that signal the presence of predatory pumpkinseed sunfish (*Lepomis gibbosus*). While growth rate, adult body size, age at maturation, and initial fecundity were not affected, peak and total fecundity were higher in the predation risk treatment. This contrasts with known life history strategies of killifish from permanent waters, which tend to reduce reproduction in the presence of predators. Although our results show that *N. furzeri* individuals are able to detect predators and respond to their presence by modulating their reproductive output, these responses only become evident after a few clutches have been deposited. Overall our findings suggest that, in the presence of a predation risk, it can be beneficial to increase the production of life stages that can persist until the predation risk has faded.

## 1 INTRODUCTION

To reduce negative effects of predation on survival and reproductive success, individual organisms may display antipredator strategies (Lass & Spaak, 2003; Stankowich & Blumstein, 2005; Stevens, 2005). These can be present in the form of specific behaviors, morphological structures, or life history traits. Such strategies may be variable within or among populations of a species (Mateo, 2007; Smith, Miner, Wiegmann, & Newman, 2009). For instance, behavioral responses such as hiding, flashing of colored body parts, or gregarious behavior have all been shown to enhance prey survival rates (Daly, Behrends, Wilson, & Jacobs, 1992; Edmunds, 1974; Sih, 1987; Stevens, 2005). Morphological features include for instance camouflage, aposematic coloration, Batesian mimicry, and protective spines or plates (Edmunds, 1974; Hoogland, Morris, & Tinbergen, 1956; Skelhorn, Rowland, & Ruxton, 2010; Stevens & Merilaita, 2009). In terms of life history responses, predation pressure has been shown to induce shifts in age at maturity, fecundity, and growth (Reznick, Butler Iv, & Rodd, 2001; Stoks, Govaert, Pauwels, Jansen, & De Meester, 2016) to increase prey fitness. Directionality of changes may even depend on the type of predator. For instance, guppies have been shown to mature at a later age and a larger size in response to gape‐limited predators while they mature earlier and at a smaller size in response to larger predators that are able to consume guppies of any size (Reznick, 1982, 1989; Reznick & Endler, 1982).

Prey responses to predators can be subdivided into two categories. Constitutive antipredator strategies are continuously expressed in the phenotype. They are most common when predation pressure does not vary in time (Edgell Timothy, Lynch Brian, Trussell Geoffrey, & Palmer, 2009). Maintaining constitutive defenses, however, can be energetically costly. For example, investment in defensive structures may lower the energy available for reproduction (Kats & Dill, 1998). If predator presence is highly variable, it can be more cost‐efficient to only develop defenses when the predator is actually present. Such inducible strategies often offer the additional advantage that the intensity of the anti‐predator response can be adjusted depending on the perceived predation risk (David, Salignon, & Perrot‐Minnot, 2014). However, to be effective, predator presence should be detectable through cues. Fish have been shown to rely on olfactory (Dixson, Munday, & Jones, 2010; Manassa, Dixson, McCormick, & Chivers, 2013) and visual cues (Manassa et al., 2013; Pita, Moore, Tyrrell, & Fernández‐Juricic, 2015) to detect predators in their environment. In addition, defenses need to be induced as soon as possible after a predator is detected. In this context, behavioral responses are often easier to induce than profound morphological changes. Generally, activity related to foraging, exploration, and reproduction is reduced to avoid detection (Dill, Hedrick, & Fraser, 1999; Figueira & Lyman, 2007; Relyea & Auld, 2005). Although morphological responses, such as the formation of helmets or spines in water fleas (Lass & Spaak, 2003), or life history shifts, such as early or delayed metamorphosis in frogs, take time to develop, they equally serve to maximize fitness in environments with predators (Laurila, 1998).

In this study, we focus on potential responses *Nothobranchius* killifish to a predation risk. *Nothobranchius* is a genus of tooth carps (Cyprinodontiformes), found throughout eastern and southern Africa. All species are characterized by a marked sexual dimorphism, with the brightly colored males being larger than the brown females (Wildekamp, 2004). These annual fishes are adapted to survive in temporary ponds. All species are exclusive to ponds that dry out annually. A fast life cycle (Blažek, Polačik, & Reichard, 2013) enables them to generally reproduce even during short inundations. Lifespan of *Nothobranchius* killifish may be linked to the inundation length of their habitat. For instance, Terzibasi et al. (Terzibasi Tozzini et al., 2013; Terzibasi et al., 2008) report correlations between lifespan and local climatic conditions but direct links between pond hydrology and life history have not been established. The subsequent dry phase is bridged through the production of dormant eggs (Watters, 2009).

A fraction of the dormant *Nothobranchius* eggs may hatch when the habitat is inundated (Furness, Lee, & Reznick, 2015; Pinceel et al., 2015), either via rainfall, or via flooding of an adjacent river. During the wet phase, the fishes not only face time stress to complete their life cycle and produce dormant eggs before their habitat dries out, they may also be exposed to a variety of predators. Avian predation is difficult to quantify, but is considered to contribute significantly to extrinsic mortality in *Nothobranchius* populations (Haas, 1976; Reichard, Polačik, Blažek, & Vrtílek, 2014). Also large belastomid hemipteran water bugs are potent sit‐and‐wait predators, capable of consuming several individuals per week (Reichard et al., 2014). The only potential resident fish predator in *Nothobranchius* habitats is the African lungfish (Reichard et al., 2014). In habitats with several *Nothobranchius* species, some larger species could eat smaller individuals of other species. Similarly, cannibalism could occur when different cohorts are present in a population but this has not been confirmed. For flood plain ponds, the situation is more complex. Depending on the intensity of rainfall, a temporary connection may be established with water from growing rivers resulting in an influx of riverine predatory fish. In addition, depending on distance to the river, some riverine predatory fish such as the catfish *Clarias* may successfully colonize temporary ponds by moving overland. A seemingly adaptive response to riverine fish was found in *N. steinforti,* where the hatching fraction of eggs was lowered in the presence of predatory *Lepomis gibbosus* pumpkinseed sunfish, even though this species is not native to the African continent (Pinceel et al., 2015). Eggs that refrain from hatching might escape predation and hatch in a subsequent, potentially predator‐free, inundation.

While the potential for phenotypic plasticity in the postembryonic life stages of *Nothobranchius* killifish has been demonstrated (Grégoir et al., 2017; Valenzano, Terzibasi, Cattaneo, Domenici, & Cellerino, 2006; Valenzano, Terzibasi, et al., 2006; Vrtílek & Reichard, 2015), the response of juvenile and adult individuals to predation risk by riverine fish is currently unknown. In other toothcarps, maturation could be plastically accelerated at the expense of somatic growth to increase the chances of reproductive success before being predated upon (Reznick, 1990). Alternatively, maturation can also be delayed to prioritize growth to evade gape‐limited predation or as a side effect of a lowered foraging activity to minimize the risks of being detected (Belk, 1998; Gosline & Rodd, 2008; Johnson, 2001). Similarly, reproductive efforts may increase, to reproduce as much as possible before being predated (Dzikowski, Hulata, Harpaz, & Karplus, 2004) or decrease due to reduced foraging activity (Johnson, 2001). Given that *Nothobranchius* fish likely grow and reproduce as fast as possible (Cellerino, Valenzano, & Reichard, 2016), it is questionable whether predator cues could still induce them to speed up development even more or change their relative investment in growth vs. reproduction.

**Figure 1:**
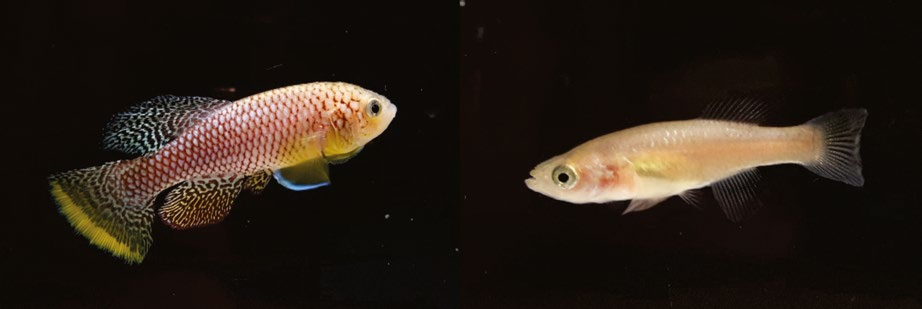
Left: a brightly colored adult male *Nothobranchius furzeri*. Right: an adult *N. furzeri* female

In this study, we focus on *Nothobranchius furzeri* as this is one of the most rapidly developing and short‐lived species (Blažek et al., 2013; Figure 1). We test whether the fast life history pace of this African killifish can still be altered by means of phenotypic plasticity in response to predator cues. For this, we exposed *N. furzeri* to visual and olfactory cues of the pumpkinseed sunfish. We hypothesize that *N. furzeri* responds to predator cues with a shift in life history. Adults are expected to either attain a larger body size to evade gape‐limited predation or stay smaller to be less conspicuous. In addition, we expect an earlier onset of reproduction rather than a delay, as the latter might be costly in a time constrained habitat and other toothcarps have been shown to be capable of early maturation in response to a predation risk (Reznick, 1990). Similarly, we expect a higher fecundity in exposed fish, so that potential reproductive output is maximized before being predated upon, as previously also suggested for guppies (Dzikowski et al., 2004). Similarly to what was found for *N. wattersi* in response to desiccation risk (Grégoir et al., 2017), we expect that any potential upregulation in maturation time or reproductive effort entails costs, such as a shorter lifespan or smaller adult body size.

## 2 MATERIAL AND METHODS

### 2.1 Fish maintenance

All fish were kept in a climate controlled room at 24°C, under a 14‐h:10‐h light:dark regime. Fish were hatched in aerated dechlorinated tap water at a temperature of 12°C, as cool water stimulates hatching (Polacik, Blazek, & Reichard, 2016). From the first day after hatching, healthy larvae were kept individually in jars filled with 250 mL dechlorinated tap water. At day five, this volume was increased to 1L. After 14 days, fish were transferred to 20L aquaria, where they were housed individually. For the first three weeks of their lives, fish were fed *ad libitum* with newly hatched *Artemia* nauplii twice daily (Ocean Nutrition, Essen, Belgium). In the fourth week, they were weaned with finely chopped *Chironomus* larvae (Ocean Nutrition). From week five onwards, they were fed *ad libitum* with frozen *Chironomus* larvae twice a day. Throughout the entire experiment, one snail (*Pomacea* spp.) was added to each aquarium to clear any excess food. All jars and aquaria were cleaned three times per week from day five onwards by suctioning all debris and replenishing three quarters of the water volume with dechlorinated tap water.

Pumpkinseed sunfish, used as predators, were housed in a large 80‐L aquarium when not co‐housed with *Nothobranchius* to impose a predation risk (see Experimental setup) and fed with frozen *Chironomus* larvae every second day.

### 2.2 Experimental setup

Each 20‐L aquarium was subdivided into four compartments: three small ones (4L) on the one side and a larger compartment (8L) on the other side (see Fig. S1). The three small compartments, housing a single *N. furzeri* individual each, were separated by an opaque barrier, preventing visual contact between the fish. In the predator exposure treatment, the large compartment housed one predatory pumpkinseed sunfish for 24 hr every second day. When adding or removing the predator, control individuals were equally disturbed by entering the predator compartment with an empty net. This compartment was separated from the three smaller ones by a transparent, perforated acrylate barrier, allowing visual, olfactory, and auditory cues to be used by the killifish to detect the presence of the predator. In total, we used 22 fish per treatment.

### 2.3 Quantified variables

We quantified key life history traits: growth, adult body size, age at maturation, early fecundity, peak fecundity, total fecundity, and lifespan. To quantify growth, fish were photographed at different ages (5, 7, 9, 19, 26, 37, 47, 58, and 79 days, respectively). For this, fish were placed in a petri dish in 1 cm of water to prevent vertical movement and photographed from above over calibrated graph paper. These pictures were then analyzed using *ImageJ* software (Schneider, Rasband, & Eliceiri, 2012). Total length at an age of 79 days was used as adult body size. Age at maturation was assessed using different criteria for males and females. Males were designated as mature when their nuptial coloration appeared (Reichard & Polačik, 2010). For the dull colored females, we recorded the age at which the first egg was produced as maturity criterion. To that end, two complementary methods were applied. First, females were provided with fine white sand as spawning substrate, allowing them to spawn eggs in the absence of males. This sand was sieved and checked daily for eggs. This was merely a safety measure as, in general, gravid females only deposit eggs when stimulated by an adult male. Therefore, starting from week six until maturity was confirmed, every fish—excluding mature males—was placed in a 1‐L jar with a bottom layer of sand together with an older, nonexperimental adult male during 30 min to stimulate potential egg deposition. Afterwards, the sand was sieved and eggs were counted. This was performed three times a week. From maturity onwards, fish were no longer provided with sand in their home tanks. Mature females were allowed to spawn with an experimental male of the same treatment three times weekly until their death. This was performed by coupling each female fish with a male for two hours in individual 1L jars with sand substrate in which eggs are buried, preventing fish from consuming newly produced eggs. Fish were coupled following a crossing scheme so that at the end of the experiment, every possible male–female combination had been made. Fecundity was decomposed in three different measures: early fecundity (total number of offspring produced in the first three weeks after maturation), peak fecundity (maximum number of offspring produced in a single week), and lifetime fecundity (total number of offspring produced). Finally, mortality was checked on a daily basis, to record lifespan.

### 2.4 Data analysis

All analyses were performed in R version 3.1.3. For growth, individual von Bertalanffy growth curves were fitted. Using the *lmer* function of the lme4 package, general linear mixed models were constructed to assess the impact of the predictor variables on von Bertalanffy growth parameters (k and L_max_, respectively). Predictor variables in the models were the predator exposure treatment (control, predator cues), sex (male, female) as well as their interaction. Besides these fixed factors, aquarium identity was added as a random factor, nested within predator exposure. This corrects for the fact that three fish were exposed to cues in separate compartments of the same aquarium. Adult body size was analyzed, with an analogous general linear mixed model and the same predictor variables. Age at maturation was analyzed separately for males and females as this response variable was scored differently. For both, a general linear mixed model was constructed with the *lmer* function. Predator exposure was included as a main effect and aquarium identity as a random factor nested within predator exposure in these models.

For all three measures of fecundity, a generalized linear mixed model was constructed using the *glmer* function of the lme4 package. A Poisson distribution was assumed, as is most appropriate for count data. Predator exposure, female adult body size, as well as their interaction were added as fixed factors to the models while aquarium identity was added as a random factor, nested within predator exposure.

The impact of predation presence on life span was analyzed by constructing a mixed effect Cox model which allows to include random effects. For this, we used the *coxme* function of the Coxme package. Sex and predator exposure as well as their interaction were included as fixed effects, and aquarium ID was added as a random effect. The *ANOVA* function of the car package was used to calculate analysis‐of‐variance tables on the constructed models.

## 3 RESULTS

### 3.1 Maturation time

**Figure 2:**
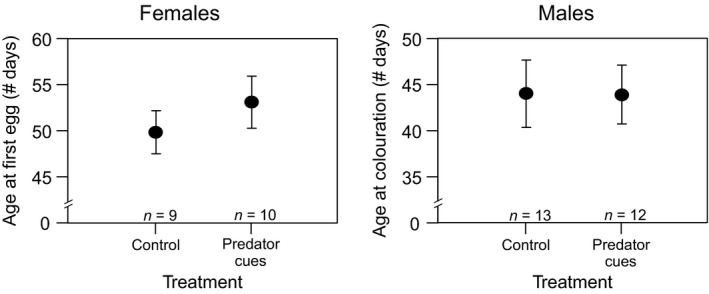
Age at maturation of *Nothobranchius furzeri* in relation to exposure to predator cues of pumpkinseed sunfish, expressed as the mean age at which the first egg was laid (females, left) or as the mean age at which the first signs of coloration were observed (males, right). Whiskers delineate the standard error

In both sexes, there was no effect of predator exposure on the age at which the fish matured (males: *F*_1,20_ = 0.001; *p *= .99; females: *F*_1,14_; *p *= .36; Figure 2). In males, the first signs of their nuptial coloration appeared, on average, at an age of 44.08 ± 3.65 days in the control group, and at an age of 43.92 ± 3.18 days in the exposed group. Females produced their first egg at an age of 49.89 ± 2.26 days and 53.2 ± 2.77 days in the control and exposed group, respectively.

### 3.2 Growth and body size

For both von Bertalanffy growth parameters and adult body size, the interaction between sex and predator exposure was not significant and therefore removed from the model. Neither prey sex, nor predator exposure significantly impacted any of the von Bertalanffy growth parameters (Table 1). Males grew significantly larger than females (36.6 ± 2.69 mm vs. 33.6 ± 2.75 mm, respectively). Predator exposure did not significantly affect adult body size (Table 1).

**Table 1:** ANOVA results based on the general linear mixed models of two von Bertalanffy growth parameters (growth parameter k and asymptotic maximum length Lmax) and adult body size. Nonsignificant interaction terms were removed from the model. Significant *p*‐values are highlighted in bold

Measure	Factor	Df/Res. DF	*F*	*p*
k	Sex	1/39	0.06	.81
	Predator exposure	1/39	0.001	.98
Lmax	Sex	1/39	0.11	.74
	Predator exposure	1/39	2.12	.17
Adult body size	Sex	1/32	9.62	**.004**
	Predator exposure	1/32	0.14	.71

### 3.3 Fecundity

**Figure 3:**
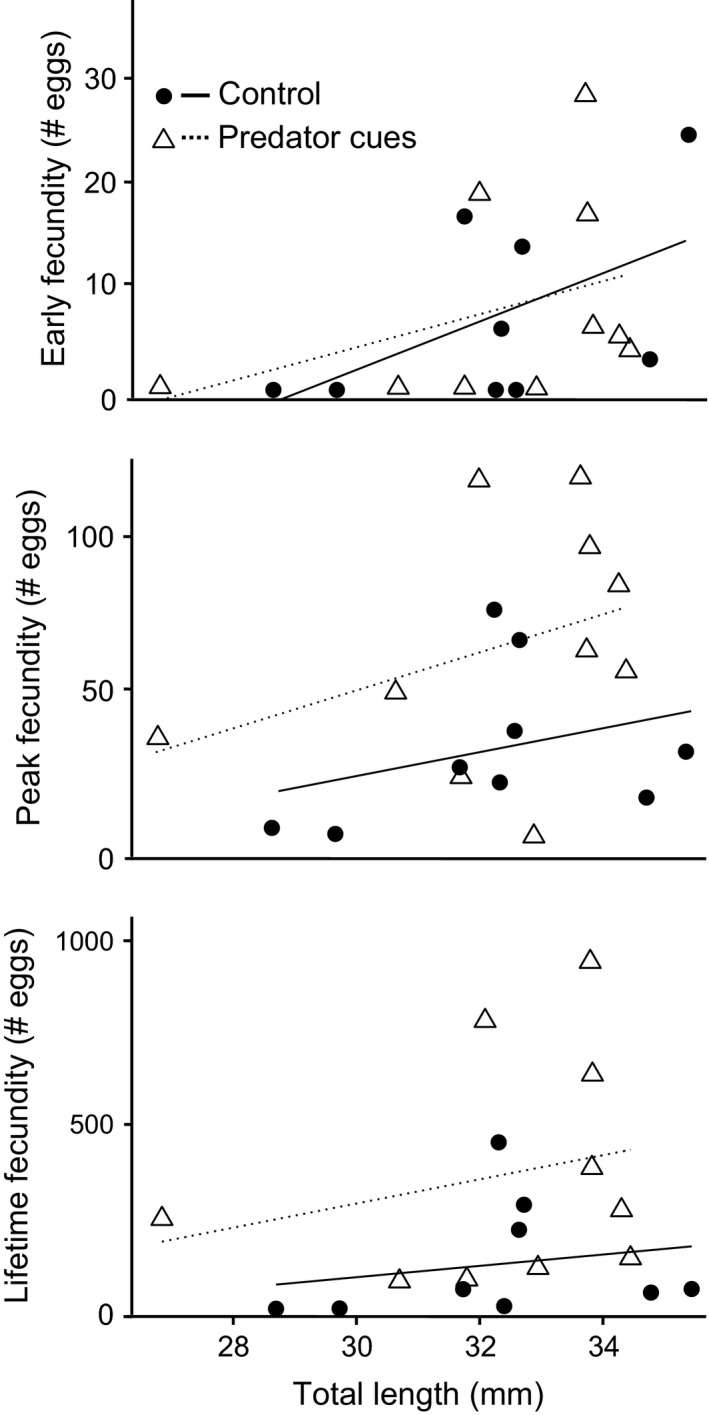
Fecundity measures of *Nothobranchius furzeri* females in relation to exposure to predator cues of pumpkinseed sunfish (circles = control, triangles = exposed) and their maximal body size (indicated by the regression line; solid = control, dashed = exposed). Number of eggs deposited the first three weeks after maturation (early fecundity, top), the maximum number of eggs produced in one week (peak fecundity, middle), or the total number of eggs produced (lifetime fecundity, bottom), all in function of body size

Early fecundity (eggs deposited in the first three weeks after maturation) was higher in larger females, but was not significantly affected by exposure to a predator (Table 2, Figure 3). Peak fecundity (the maximum number of eggs deposited in a single week) increased significantly from 34 ± 8.6 eggs to 68 ± 12.59 eggs in individuals exposed to predator cues compared to control animals, with an increasing number of eggs produced with increasing body size. Lifetime fecundity was increased in a similar fashion by predator exposure, with an increase from 136.3 ± 50.08 eggs to 341.3 ± 96.28 eggs in exposed individuals. Again, body size positively impacted the number of eggs produced. For all three fecundity measures, the interaction term between female body size and predator exposure had no significant effect and was hence removed from the final models.

**Table 2:** ANOVA results based on the generalized linear mixed models for three fecundity measures (early = eggs deposited in the first three weeks after maturation, peak = the maximum number of eggs deposited in a single week, and lifetime = the total number of eggs deposited) explained by female body size and predator exposure. Note that for all three measures, a Poisson distribution was assumed. Nonsignificant interaction terms were removed from the model. Significant *p*‐values are highlighted in bold

Measure	Factor	χ²_1,12_	*p*
Early	Female body size	17.01	**<.001**
	Predator exposure	3.20	.07
Peak	Female body size	11.06	**<.001**
	Predator exposure	5.71	**.017**
Lifetime	Female body size	156.62	**<.001**
	Predator exposure	5.93	**.015**

### 3.4 Survival

**Figure 4:**
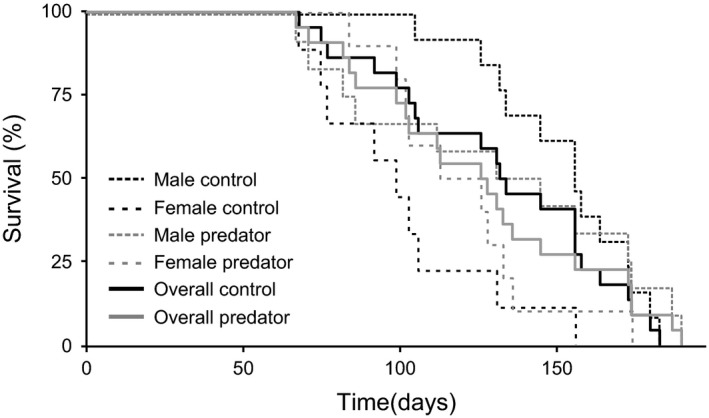
Survival curves showing the proportion of surviving *Nothobranchius furzeri* individuals in relation to exposure to predator cues of pumpkinseed sunfish. Thick lines represent the overall response including fishes of both sexes. Dashed lines show the response subdivided for the two sexes separately

Sex had a significant effect on survival according to the mixed Cox model (z_1,41_ = −3.18, *p *= .002; Figure 4) with males living longer than females (average lifespan of 142.4 ± 7.4 days vs. 110.8 ± 6.4 days, respectively). Predator exposure had no effect (z_1,41_ = −1.31, *p *= .19). A nonsignificant predation x sex interaction terms was removed from the final model.

## 4 DISCUSSION

For all organisms that breed in temporary ponds, pond drying imposes time‐constraints on maturation, reproduction, or both. Before the end of an inundation, amphibians and many aquatic insects need to complete metamorphosis to escape, whereas others such as killifish and many crustaceans need to produce dormant life stages to survive the drought *in situ* (Williams, 2006). In *Nothobranchius* killifish, lifespan may be linked to the typical lengths of the inundations that they experience in their local habitat (Terzibasi Tozzini et al., 2013; Terzibasi et al., 2008). In addition, *Nothobranchius* may still retain some flexibility in dealing with impending pond drying as it has been shown that desiccation risk simulated by means of a drop in water level resulted in a plastic increase in egg deposition at the cost of a shorter lifespan (Grégoir et al., 2017). In this study, we test whether *Nothobranchius* killifish can produce similar plastic life history responses when exposed to predation risk rather than desiccation risk. Overall, we found no indication that these fishes can accelerate or decelerate their development toward maturation in response to a predation risk. However, our results do suggest higher reproductive output when cues signal a predation threat.

A change in maturation time in response to a perceived predation risk has been observed in a number of taxa, with maturation at a younger age in, for example, *Daphnia* (Stoks et al., 2016) or *Culex* mosquito larvae (Silberbush, Abramsky, & Tsurim, 2015), or delayed maturation in sunfish (Belk, 1998), *Rana* tadpoles (Laurila, 1998; Laurila & Kujasalo, 1999), or other amphibians (Relyea, 2007). In *Nothobranchius* killifish, however, we detected no plastic response in maturation time in either direction. Potentially, the cue was not correctly identified as a predation threat, given that the imposed predator, pumpkinseed sunfish, does not occur in the natural range of *Nothobranchius*. However, this is unlikely as eggs of congeneric species (Pinceel et al., 2015) do detect and respond to the cues of this predator and so do the older life stages of the species in this study by increasing their fecundity as indicated by our results. Alternatively, the absence of such a response in these fishes could be due to the time stress that is inherent to their temporary habitat. First of all, *Nothobranchius* fishes are likely positively selected to already attain maturity as soon as possible, to reproduce before desiccation. Hence, any further metabolic acceleration to mature at an even younger age might be physiologically unfeasible. In support, the congeneric *N. wattersi* also shows no developmental acceleration in response to a drop in water level (Grégoir et al., 2017), suggesting that such an acceleration might indeed be impossible. Secondly, as a consequence of the time stress experienced in the natural habitat, all individuals probably mature as soon as possible, even in the presence of a predator. As the growing season is lime limited and the risk of early desiccation can be high, any delay in maturation may be maladaptive. Delayed metamorphosis as a side effect of reduced foraging rates in response to a predation threat observed in *Rana* tadpoles is no longer observed when complemented with a risk of desiccation (Laurila & Kujasalo, 1999), indicating that the latter stressor has priority. Furthermore, modeling of population growth rates of crustaceans from temporary pools, which have a similar life‐cycle to that of *Nothobranchius* killifish, shows that any delay in maturation has strong negative effects on long‐term demographics (Pinceel, Vanschoenwinkel, Brendonck, & Buschke, 2016)(Pinceel et al., 2016).

Besides a lack of response in maturation time, we did not record differences between control and exposed fish in growth rate or final body size. Both increased and decreased growth rates have been observed as nonconsumptive effects of predation on prey (Peacor, 2002; Peckarsky, Taylor, McIntosh, McPeek, & Lytle, 2001). Faster growth rates are often attributed to the thinning effect of predators, increasing the amount of resources available for survivors. Given that actual predation was excluded from the setup and that all individuals received equal amounts of food, such a thinning effect is not applicable here. A larger body size, even when this is a side effect of increased resource availability due to thinning effects, can help to escape predation by gape limited predators (Day, Abrams, & Chase, 2002; Urban, 2007). However, in this context, where maturity has to be reached as fast as possible, a redirection of energy to something other than maturation appears to be unlikely. Slower growth rate in prey organisms can be a side effect of hiding behaviour and the correlated decreased food intake (Abrams & Rowe, 1996; Rowe & Ludwig, 1991; Urban, 2007). Although speculative, it seems likely that *Nothobranchius* fish cannot afford to hide in a natural habitat because it needs to feed intensively to be able to mature and reproduce prior to pond desiccation.

Later in life, *Nothobranchius* killifish did respond to predation risk in our experiment. Whereas early fecundity was not affected by a predation threat, the maximal peak in fecundity was doubled relative to that of control animals. The reproductive increase in response to a predation threat contrasts with findings in many other organisms, including the riverine killifish *Rivulus hartii*, where all activity, including reproductive effort, are lowered to reduce the probability of being detected by a predator (Creel, Christianson, Liley, & Winnie, 2007; Fraser & Gilliam, 1992; Zanette, White, Allen, & Clinchy, 2011). Yet, again, the time‐constraint imposed by their habitat probably ensures that in the studied population, any decrease in fecundity on the short term cannot be compensated by a lengthening of the reproductive period, as would be possible for organisms from permanent habitats. Whereas the latter might achieve a higher lifetime reproductive output by lowering current reproductive efforts, this might be maladaptive for time‐constrained organisms. As such, *Nothobranchius* killifish seem to apply a largely opposite strategy compared to *R. hartii*, producing as many offspring as possible before being predated. Such a response has also been observed in *Daphnia* (Stibor, 1992; Stoks et al., 2016) and could be an adaptive strategy in a temporary environment. Another element that makes this strategy highly effective is the fact that *Nothobranchius* eggs, once produced, hatch only after a desiccation event that effectively removes (most) fish predators (Cellerino et al., 2016). This ensures that the offspring, when buried in the relative safety of pond sediment, can effectively “wait” until the predation risk has faded.

Increasing fecundity should, however, always come at a cost (Stearns, 1992). Otherwise, control animals that were not exposed to predator cues, would have no reason to reproduce less than their predator exposed counterparts. Reproductive output could, for instance, trade‐off with lifespan. Yet, no such trade‐off was found between exposed and control females, as fish in both treatments died at the same rates. This contradicts earlier findings on *N. wattersi* that were exposed to cues that signal desiccation risk (Grégoir et al., 2017), but is in line with the results of previous studies on *N. furzeri*, where female egg deposition was shown to decrease in the absence of males (Graf, Cellerino, & Englert, 2010). As to why such differences are found between studies remains unclear. The existence of species‐specific trade‐offs seems unlikely at first sight, especially given the high ecological similarity between different *Nothobranchius* species (Polačik, Harrod, Blažek, & Reichard, 2013). Yet, growing evidence suggests that trade‐offs might differ substantially, even between such closely related species (Messina & Fry, 2003). Alternatively, trait covariances might be expressed differentially across environmental contexts (Messina & Fry, 2003; Messina & Slade, 1999). The reproductive increase in response to a drop in water level seemed to be at the expense of lifespan (Grégoir et al., 2017), while the costs of a similar response to predator cues appear to be different. With respect to potential trade‐offs between reproductive output and lifespan, it must be noted that most laboratory experiments are conducted under conditions that are highly divergent from the natural situation. For instance, males likely outlive females in this experimental setting because of the difference in energetic investment compared to females. Male reproductive investment in the wild mainly involves male–male competition, which was eliminated here, whereas female investment involves the energetically costly production of eggs. Although not tested here and not found in response to a drop in water level (Grégoir et al., 2017), the number of offspring can be increased without an extra energy investment in reproduction by simply lowering the energetic investment per offspring individual. That way, the increase in offspring quantity might have been traded off with a lower offspring quality (Smith & Fretwell, 1974). Alternatively, exposed individuals may have had a lower body mass, despite being equally large as control individuals, or may have invested less in immune functioning. Such effects on survival may only become apparent in a more natural setting with more food stress and exposure to pathogens. Another cost may be related to an increased predation risk by other predators. In the wild, there is always the substantial risk of avian or insect predation (Reichard et al., 2014), as birds and hemipterans are highly mobile and are expected to impose a predation threat in nearly all inundated *Nothobranchius* habitats. Feeding activity of control animals might be restrained as a consequence of such predation risks. For individuals exposed to riverine fish, which may impose a more acute predation risk, individuals may have increased their feeding activity, thereby potentially also increasing their vulnerability to avian or bug predation. Based on our experiment, however, we have no indication that individuals exposed to predator cues ate more than control individuals fed equal amounts of food. Still, overall our results do show that *Nothobranchius* fish are able to detect and respond adaptively to predator presence by modulating reproductive output, but that these responses only become evident after a few clutches have been deposited.

## CONFLICT OF INTEREST

None declared.

## AUTHOR CONTRIBUTIONS

AFG, LB, TP, and BV designed the experiment. AFG, ESJT, and CP performed the practical work. AFG, ESJT, and BV analyzed the data. AFG and BV drafted the text. All authors revised it critically and significantly improved the text. All authors agree to the final version of the text.
